# Blocking premature reverse transcription fails to rescue the HIV-1 nucleocapsid-mutant replication defect

**DOI:** 10.1186/1742-4690-8-46

**Published:** 2011-06-17

**Authors:** James A Thomas, Teresa L Shatzer, Robert J Gorelick

**Affiliations:** 1 AIDS and Cancer Virus Program, SAIC-Frederick, Inc., NCI at Frederick, Frederick, MD 21702, USA

## Abstract

**Background:**

The nucleocapsid (NC) protein of HIV-1 is critical for viral replication. Mutational analyses have demonstrated its involvement in viral assembly, genome packaging, budding, maturation, reverse transcription, and integration. We previously reported that two conservative NC mutations, His23Cys and His44Cys, cause premature reverse transcription such that mutant virions contain approximately 1,000-fold more DNA than wild-type virus, and are replication defective. In addition, both mutants show a specific defect in integration after infection.

**Results:**

In the present study we investigated whether blocking premature reverse transcription would relieve the infectivity defects, which we successfully performed by transfecting proviral plasmids into cells cultured in the presence of high levels of reverse transcriptase inhibitors. After subsequent removal of the inhibitors, the resulting viruses showed no significant difference in single-round infective titer compared to viruses where premature reverse transcription did occur; there was no rescue of the infectivity defects in the NC mutants upon reverse transcriptase inhibitor treatment. Surprisingly, time-course endogenous reverse transcription assays demonstrated that the kinetics for both the NC mutants were essentially identical to wild-type when premature reverse transcription was blocked. In contrast, after infection of CD4+ HeLa cells, it was observed that while the prevention of premature reverse transcription in the NC mutants resulted in lower quantities of initial reverse transcripts, the kinetics of reverse transcription were not restored to that of untreated wild-type HIV-1.

**Conclusions:**

Premature reverse transcription is not the cause of the replication defect but is an independent side-effect of the NC mutations.

## Background

The nucleocapsid (NC) protein of HIV-1 functions throughout the viral replication cycle, from involvement in assembly and genomic RNA (gRNA) packaging as part of the Gag protein (Pr55), to facilitating reverse transcription as a mature protein (p7). The mechanisms behind NC's ability to perform these roles have been extensively investigated both *in vitro *and in cell culture as detailed in the following reviews [1-8].

The role of NC in reverse transcription has been investigated in considerable detail using a number of excellent *in vitro *systems. Because of these thorough studies, we know that NC can facilitate the tRNA^lys3 ^annealing to the primer binding site [9-11], dramatically enhance the efficiency of minus-strand and plus-strand transfer events [12-19], prevent self-priming (a suicidal reaction) [13,15,18,20,21], and enhance the processivity of reverse transcription [22-25]. In addition to reverse transcription, NC has also been demonstrated to enhance coupled integration events *in vitro *[26]. The fact that NC can assist in all of these processes directly proceeds from its properties as a nucleic acid chaperone, which means that NC assists nucleic acids to find the most thermodynamically stable arrangement resulting in maximum base pairing [1,2]. Although the general properties of NC as a nucleic acid chaperone were observed many years ago *in vitro *[17,27], the mechanics of how these properties govern NC's actions during reverse transcription is still being elucidated.

We have been interested in examining how NC mutations affect reverse transcription in virions and infected cells. Two particular mutants of HIV-1, NC_H23C _and NC_H44C_, have proven to be of great interest in that although the amino acid alterations are functionally conservative with respect to zinc binding, genome packaging, and virion assembly, the resulting viruses are replication defective [28-30]. Our initial studies revealed an apparent defect in viral DNA (vDNA) stability and integration after infection [31]. After a more detailed kinetic analysis, we were able to directly demonstrate that integration efficiency was severely impaired for both of these mutants [32]. Intriguingly, these data also suggested that these NC mutations appear to cause reverse transcription to initiate much earlier than in wild-type infections. When we examined the nucleic acids present in NC-mutant virions prior to infection, we found that they actually contained a significant amount of vDNA (~1,000-fold more than WT [33]); virtually every particle had initiated reverse transcription, and so this process is apparently occurring prematurely in the viral replication cycle. Similar results have also been reported by another group with these and other HIV-1 NC-mutant viruses [34,35]. The exact cause and the significance of this premature reverse transcription are unknown [33,36].

We hypothesized that premature reverse transcription alone may have been sufficient to block replication of these viruses. Therefore, we attempted to block premature reverse transcription in the NC mutants using reverse transcriptase inhibitors (RTIs) rather than reverse transcriptase (RT) active site mutations. This choice was made because arresting reverse transcription with inhibitors is potentially reversible, which would enable us to assess how well blocking premature reverse transcription affects viral replication. Additionally, we have observed that active site point mutations in RT can cause unwanted alterations in Gag processing (data not shown). A previous study demonstrated the feasibility of reducing intravirion DNA by greater than 97% by the addition of 50 μM Nevirapine (NVP); treatment with 50 μM azidothymadine was only able to reduce intravirion DNA by 75% [35].

## Results

### Reverse transcriptase inhibitors prevent infection and can be effectively removed from virus preparations

Initial experiments were performed to determine the necessary concentrations of RTIs to use and we found that a single inhibitor was insufficient to block the levels of premature reverse transcription that the NC mutations were causing (data not shown). Virtually every NC-mutant virus particle contains minus-strand strong-stop DNA [33], which is extremely difficult to prevent because it is much more difficult to inhibit the synthesis of short reverse transcripts (i.e., minus-strand strong-stop DNA) [37] required for these studies. In contrast, viral replication can be blocked if the synthesis of the full-length reverse transcript is stopped at almost any point. We ultimately found that in order to effectively stop premature reverse transcription, we needed to add very high concentrations of two different RTIs to cells, immediately before transfection of proviral plasmids: 1.0 mM Tenofovir (PMPA) and 50 μM NVP. These two drugs target RT differently; PMPA is a nucleotide reverse transcriptase inhibitor (NRTi) that must be incorporated into the nascent DNA while NVP is a non-nucleoside reverse transcriptase inhibitor (NNRTi). The concentrations of each inhibitor required to completely prevent intravirion DNA synthesis were more than 1,000-fold higher than their IC_50 _levels in cell culture (PMPA: IC_50 _= 0.1-0.6 μM [38], NVP: IC_50 _= 40 nM [39]).

**Figure 1:**
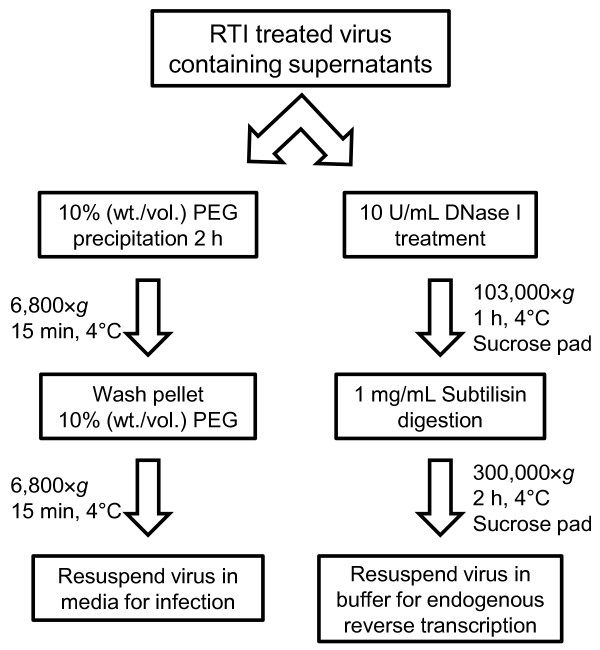
**Methods to remove RTIs from virus preparations**. Schematic of the two methods used to remove RTIs from virus preparations. The RTIs were removed so that they did not inhibit downstream assays to assess viral function when premature reverse transcription was blocked. In both methods aspiration was used to remove the supernatant after centrifugation (see the Methods section for details). The method on the left was used to i) maintain competent Env proteins on the surface of virions and ii) limit mechanical stress on virions for subsequent infection analyses. The method on the right uses DNase I treatment to remove extra-virion plasmid DNA contamination with subsequent subtilisin digestion to ensure that the DNase I is completely removed prior to lysis of the virions, and qPCR analysis of intravirion DNA and endogenous reverse transcription assays [33].

However, our investigations required determining the properties of virions after premature reverse transcription had been blocked, so we developed two different methods (Figure 1) to remove excess RTIs from virus preparations once particles were released from the producer cells and premature reverse transcription could no longer occur. Key to both of these methods is the collection of the virus particles for complete media replacement, which reduces the concentration of RTIs to levels far below what would interfere with reverse transcription. For subsequent infectivity experiments, we precipitated virus from culture supernatants with polyethylene glycol (PEG 8000) at 4°C (Figure 1, left). In contrast, for subsequent assessment of intravirion DNA levels and endogenous reverse transcription assays, we used our previously reported protocol for preparing virions (Figure 1, right; [33]); this rigorous protocol was found to be essential for removal of extra-virion contaminating plasmid DNA to enable accurate determinations of intravirion DNA levels [33]. However, virus treated by the latter method, which entails subtilisin digestion to inactivate the DNase I prior to lysing the virions cannot be used for infectivity assays as all membrane surface proteins, including Env, are digested [40].

**Figure 2:**
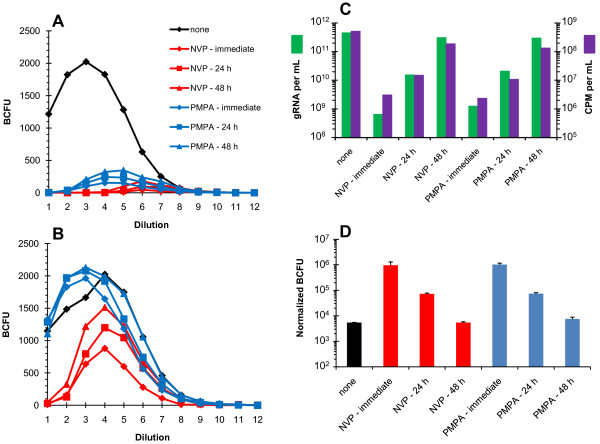
**RTIs can be effectively removed from virus preparations**. Env^(-) ^VSV-G pseudotyped WT HIV-1 expressed from 293T cells transfected in the absence (black), or presence of 1.0 mM PMPA (blue) or 50 μM NVP (red), is assayed for limiting-dilution infectivity on TZM-bl cells (panels A, B and D). The numbers on the X-axes (panels A and B) represent the dilution series, each step being a 3-fold serial dilution starting with 0.1 mL of undiluted infectious supernatant (Dilution 1). For each drug treatment, RTIs were added either immediately before transfection, 24, or 48 h after transfection as indicated in the legend at the right of panel A. Blue colony forming units (BCFU) were tallied 48 h after the infectivity experiments were started [41]. Panel A shows the titer of infectious supernatants assayed without PEG precipitation of virus. Panel B shows the titer of infectious supernatants after PEG precipitation to remove RTIs from the virus stock (see legend from panel A). Panel C shows the yield of WT virus from each transfection condition (with or without drugs) measured using either qRT-PCR (quantifying gRNA per mL, green) or exogenous-template RT assays (measuring RT activity in counts per minute of [^32^P]-TMP incorporated per mL [CPM per mL], purple). Panel D shows the titer of the PEG-precipitated WT virus, with the treatments (indicated at the bottom) normalized for the gRNA present in the starting supernatant (i.e., corrected for dilution). Values are expressed as normalized blue cell forming units (BCFU) and represent the averages from at least three dilutions (error bars indicate standard deviations).

Identifying effective methods for removal of RTIs was initially performed using the VSV-G pseudotyped HIV-1 system that we previously employed [33]. Figure 2 compares single-round TZM-bl infectivity over a serial dilution series [41] of untreated or RTI-treated VSV-G pseudotyped NC_WT _virus preparations without (panel A) or with (panel B) PEG precipitation. It is important to note that the titer of untreated virus (black line) is the same, whether the virus was PEG precipitated or not. However, the titers of viruses treated with either NVP (red lines) or PMPA (blue lines) are much lower if the RTIs are not removed (compare panels A and B, red and blue lines). NVP appears to be more difficult to remove than PMPA as the peak in PMPA-treated viruses occurs at a lower dilution than the peak in NVP-treated virus. This may be due in part to their different modes of action (NRTi vs NNRTi) so that the dATP present in the infected cells competes with any remaining unincorporated PMPA in the preparations. This difference also correlates with the relative effective concentrations of the two drugs (PMPA is effective in the μM range while NVP is effective in the nM range). Interestingly, because PMPA is a chain terminator, it functions by being incorporated into the nascent vDNA and thus would not be affected by reducing its concentration in the media. However, it has been shown that WT RT has the ability to excise nucleosides, including PMPA *in vitro *[42,43] and NC can facilitate excision processes, possibly by stabilizing RT on the nucleic acid template [42,44]. As will be shown in our assays below, removal of RTIs is essentially complete.

The fact that the wild-type virus used for the experiments in Figure 2 was VSV-G pseudotyped demonstrates another effect of the RTI treatment. Pseudotyping HIV-1 boosts the infectious titer of the virus produced in part by increasing the total number of virus particles (Figure 2C). These additional particles are the product of VSV-G pseudotyped virus infecting the transfected cells, which we showed previously could be inhibited by PMPA treatment of the transfected cell culture [33]. If one compares the yield of virus as a function of the treatment, one sees that the amount of virus produced decreases the earlier RTIs are added during the transfection (Figure 2C). In this chart, virus yields are determined using either quantitation of genomes by qRT-PCR or exogenous-template RT activity. Importantly, these two assays are in excellent agreement, which shows that the RTIs have been effectively removed and do not significantly affect the exogenous-template RT activity. For the majority of subsequent experiments, gRNA quantitation is used, because it is the most relevant for determining the efficiency of reverse transcription; vDNA results are normalized on a per genome basis throughout. The later RTIs are added during the transfection, the closer the virus yield approaches that of the untreated virus so that if RTIs are added 48 h after the DNA-precipitate is applied to the 293T cells, there is essentially no effect on virus yield. We conclude that immediate addition of RTIs to the transfected cells inhibits VSV-G mediated reinfection completely because virus yield is no different from that obtained from transfections without VSV-G (see below).

While addition of RTIs to transfected cells at earlier times decreases virus yields, we observed a corresponding increase in the infectivity per virion (Figure 2D). When RTIs are present from the immediate onset of the transfection, the infectivity per particle is approximately 180-fold higher than virus produced without RTIs (compare black bar with red and blue "immediate" bars). If RTIs are added 24 h after the transfection, the infectivity per particle is only 13-fold higher. Finally if RTIs are added 48 h post transfection, the infectivity per particle is nearly the same as virus produced without RTI exposure (Figure 2D). The decrease in relative infectivity is likely due to an accumulation of defective genomes (from the VSV-G pseudotyped wild-type virus reinfection of the transfected cells mentioned above) producing non-infectious particles because the reverse transcription process is inherently error-prone [45]. We know from previous studies that in this system a replication cycle occurs every 24 h [46], thus virions have undergone 2 rounds of replication while being generated, and genomes are no longer transcribed solely from transfected plasmids.

### RT inhibitors can block premature reverse transcription

For the remainder of this study we chose to use non-pseudotyped, Env^(+) ^HIV-1 for several reasons: i) so we do not need to be concerned with reinfection of transfected cells with the wild-type virus (without RTI treatment) and ii) it was noted previously that VSV-G pseudotyped NC-mutant HIV-1 did not undergo this amplification since the NC mutants are replication defective, thus there will not be the tremendous difference in the numbers of particles produced between VSV-G pseudotyped NC-mutant and wild-type HIV-1 that was reported previously [33]. This makes comparisons of results between untreated and RTI-treated samples more straightforward.

**Figure 3:**
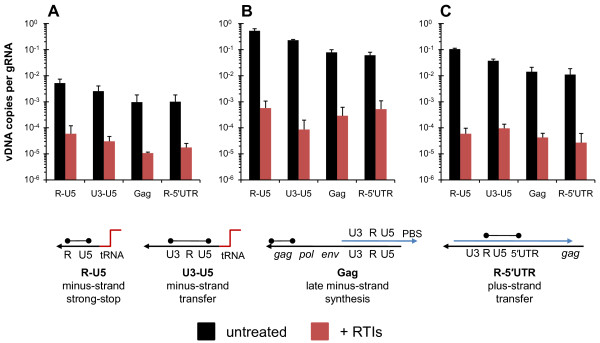
**Premature reverse transcription can be blocked**. HIV-1 was expressed from 293T cells transfected either in the absence (black bars) or presence (red bars) of RTIs. Virus was harvested and treated with DNase I and subtilisin, as described in the Methods section (Figure 1, right). Quantities of intravirion DNA were then measured by qPCR using the reverse transcription intermediate targets [31] indicated at the bottom of the figure (tRNA, red line; minus-strand DNA, black line; plus-strand DNA, blue line; target sequences indicated by the black dumbbells). The quantities expressed are the ratio of vDNA to gRNA. Panel A shows wild-type virus, panel B shows NC_H23C _virus, and panel C shows NC_H44C _virus. Values plotted are the means and the errors bars are the standard deviations from two separate experiments.

We transfected 293T cells cultured in the presence of both PMPA and NVP with NC-mutant and wild-type proviral plasmids and changed the media after 24 h, adding fresh RTIs to maintain concentrations as high as possible. We harvested virus 24 h later, treated with DNase I and subtilisin to remove extra-virion contaminating plasmid DNA (Figure 1, right), and measured intravirion DNA by quantitative PCR (qPCR) to assess the levels of minus-strand strong-stop (R-U5), minus-strand transfer (U3-U5), late minus-strand synthesis (Gag) and plus-strand transfer (R-5'UTR) targets, and also gRNA as previously described [33]. Figure 3 shows that using this method we could quite significantly (>99.9%) reduce intravirion R-U5 DNA in the NC mutants to levels below those observed for untreated wild-type virus (compare red bars in panels B and C, with black bars in panel A). When one compares the quantities of intravirion DNA per gRNA, between untreated and RTI treated samples, there is a 60- to 90-fold reduction of intravirion DNA in WT virions (panel A), a 120- to 2,600-fold reduction in NC_H23C _virions (panel B), and a 340- to 1,800-fold reduction in NC_H44C _virions (panel C), depending on the vDNA target.

After blocking premature reverse transcription, levels of intravirion DNA per gRNA are very similar between wild-type and the NC mutant virions (compare red bars between panels A with B or C [i.e., NC_H23C_:NC_WT _= 3- to 30-fold difference or NC_H44C_:NC_WT _= 1- to 4-fold difference, respectively, depending on the vDNA species]).

### Blocking premature reverse transcription has no effect on infectious titer of viruses

**Figure 4:**
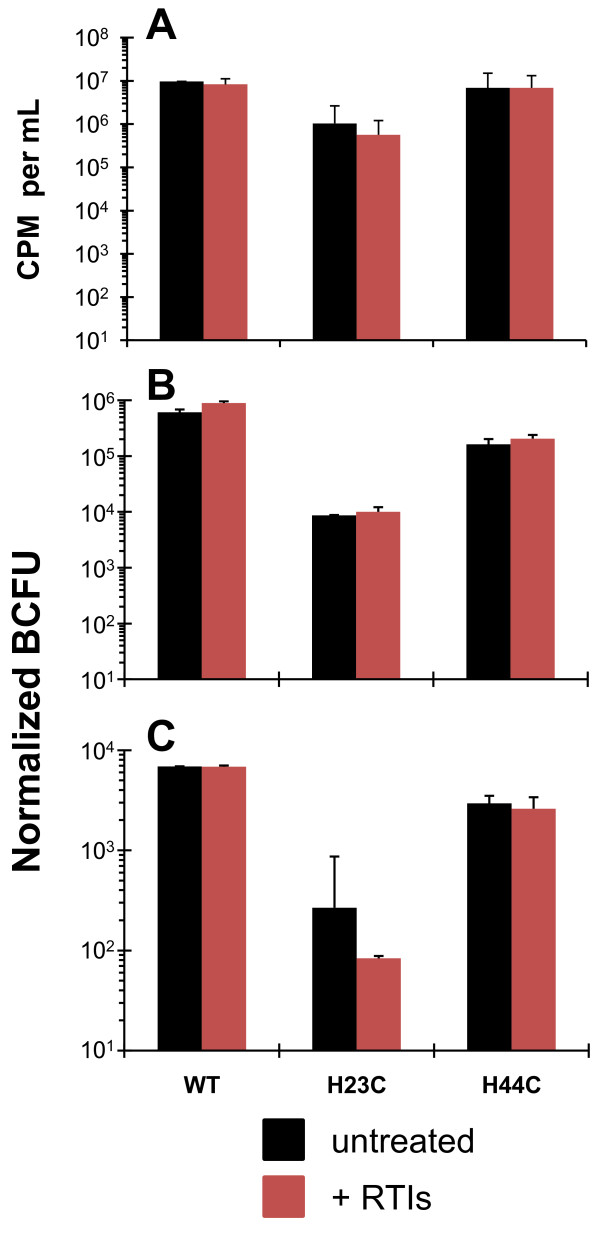
**Titer of NC mutant viruses is unchanged if premature reverse transcription is blocked**. Results from HIV-1 produced either in the absence (black bars) or the presence (red bars) of RTIs are presented. All viruses produced were PEG precipitated, not just those treated with RTIs for direct comparison. Panel A shows the yields of viruses from transfections, with and without RTI treatment based on average exogenous-template RT activities (in counts per minute of [^32^P]-TMP incorporated per mL [CPM per mL]; error bars are the standard deviation from duplicate samples). Panels B and C are from two independent transfection-infection experiments and each displays the titers of viruses measured using TZM-bl cells, determined from 3-fold serial dilutions [41]. The titer was corrected for dilution and input virus as determined by exogenous-template RT activity and expressed as "Normalized BCFU" from the means of at least 3 dilutions (error bars represent the standard deviation).

Figure 4 displays the efficacy of PEG precipitation on removing RTIs from NC_mutant _and NC_WT _virus preparations. Figure 4A shows the yield of viruses produced in the absence or presence of RTIs, expressed as exogenous-template RT activity (in CPM per ml). One can see that the RT activities are slightly lower in preparations of viruses generated in the presence of RTIs. The ~2-fold difference here (with non-VSV-G pseudotyped viruses) is significantly less than the ~1000-fold difference between untreated and RTI treated samples observed with the VSV-G pseudotyped NC_WT _virus (Figure 2C), which again has to do with the prevention of the reinfection of transfected cells using VSV-G pseudotyped virus discussed above. Thus RTI treatment does not appreciably decrease the amount of virus produced from cells. Figure 4 also shows the titers of viruses prepared in the presence or the absence of RTIs from two separate transfection/infection experiments (panels B and C). These viruses were PEG precipitated (Figure 1, left) to remove the RTIs. Critically, the titer of wild-type virus is completely unchanged whether the virus is prepared in the absence (black bars) or presence (red bars) of RTIs, firmly establishing that we can effectively remove RTIs from virus preparations. In the case of the NC_H23C _and NC_H44C _viruses, we see that blocking premature reverse transcription using RTIs had no significant effect on infectious titers (Figure 4B and 4C); importantly, infectivity was not restored to wild-type levels upon RTI treatment with the NC mutants. The relative difference in the titers of NC_WT _to that of the NC_mutants _is what we normally see when viruses are not PEG precipitated ([28]; data not shown).

### NC_mutants _display wild-type kinetics during endogenous reverse transcription

We established that blocking premature reverse transcription did not relieve the infectivity defect; therefore, we investigated the reverse transcription efficiency of the NC-mutant and wild-type viruses using an endogenous reverse transcription assay. As before (Figure 3; [33]) we used our qPCR system to measure the quantities of each reverse transcription intermediate and then normalized the vDNA quantities to the amount of gRNA present at the initiation of the assay to determine the efficiency of conversion of gRNA to reverse transcripts. We prepared the viruses either in the presence or the absence of RTIs, and then treated the viruses with DNase I and subtilisin (Figure 1, right) to remove not only contaminating extra-virion DNA, but the RTIs as well. Each virus preparation was then divided into 7 equal aliquots to examine an endogenous reverse transcription time course.

**Figure 5:**
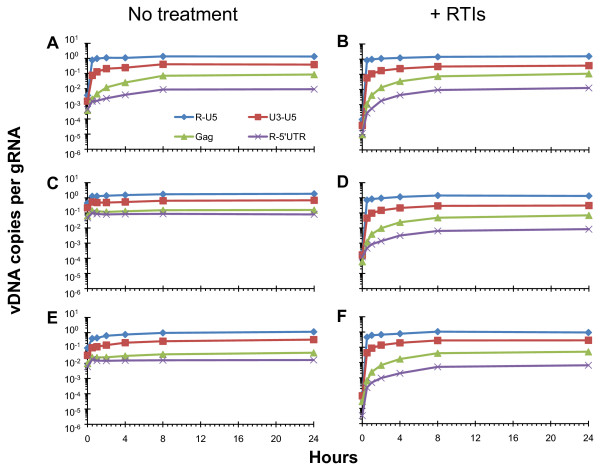
**NC mutant viruses display wild-type endogenous reverse transcription kinetics when premature reverse transcription is blocked**. Results of endogenous reverse transcription assays performed with virus generated either in the absence or presence of RTIs as indicated at the top of the figure. Virus was prepared using sequential DNase I + subtilisin treatment as described (Figure 1, right). At each time point indicated, a sample of virus was taken and the vDNA was isolated and quantitated. Values were then divided by the number of genomes present at the start of every endogenous reverse transcription reaction to display the quantity of vDNA as a fraction of available genomes. Panels A, C, and E show endogenous reverse transcription time courses using viruses (WT, NC_H23C_, NC_H44C_, respectively) prepared without RTIs while panels B, D, and F show time courses with viruses (WT, NC_H23C_, NC_H44C_, respectively) prepared in the presence of RTIs. These results are from a representative experiment. The legend for the vDNA species measured is indicated at the bottom of panel A and the bottom of Figure 3 shows schematics of the pertinent vDNA target sites.

Figure 5 shows the results of these experiments comparing the amount of each vDNA species measured as a function of time. Panels A and B show endogenous reverse transcription activity from wild-type virus prepared in the absence or the presence of RTIs, respectively. As observed above (Figure 3A) wild-type virus prepared using the RTI treatment results in a decrease of the already low levels of intravirion DNA by approximately 2 logs. This decrease in background actually enables a more accurate determination of endogenous reverse transcription activity. When Figures 5A and 5B are compared, one can see that although the kinetics of the reactions are similar, the formation of each of the measured reverse transcription products is much more efficient. The final quantities of each intermediate are the same, independent of the presence or absence of RTIs, while the initial quantities are 2-logs lower in virus prepared with RTIs. Closer inspection of Figure 5B shows several important details. Synthesis of R-U5 is very rapid, and every copy of gRNA gives rise to 1 copy of R-U5 vDNA. Synthesis of U3-U5 is also fast, although quantities continue to accumulate until 8 h into the reaction, when approximately one third of genomes have progressed to maximal U3-U5 vDNA levels. Gag targets are noticeably slower in production, with a more gradual increase to maximum quantities occurring 24 h into the reaction when approximately one tenth of genomes have progressed to generate maximal Gag vDNA. Finally, synthesis of R-5'UTR vDNA is the slowest with approximately 1 in 80 genomes being reverse transcribed at 24 h.

Examination of the quantities of vDNA present in the NC_H23C_- (Figure 5C) and NC_H44C_- (Figure 5E) mutant viruses prepared without RTIs reveals a small increase over the time course of the reaction (~4-10-fold for R-U5 DNA). As we reported previously [33], these NC mutants do not have significant endogenous reverse transcription activity, likely due to the lack of available gRNA template because of the premature reverse transcription that has taken place during production of the mutant viruses. However, when we prevent premature reverse transcription using the RTI treatment, we see that both NC_H23C _(Figure 5D) and NC_H44C _(Figure 5F) exhibit strong endogenous reverse transcription activity. For each of the vDNA species examined, the kinetics and efficiency of formation are virtually identical to what we observed with wild-type (Figure 5B), with a ~10,000-fold increase in R-U5 vDNA copies over the time course. Therefore these NC mutants are not defective in any detectable way for reverse transcription that takes place within virions. In addition, this assay demonstrated the likelihood that any PMPA incorporated when the viruses were generated was effectively removed, as the efficiencies and kinetics of this reaction are the same as wild-type. It is possible that some fraction of nascent transcripts still contains incorporated PMPA and was not extended, but this population was not apparent in this assay.

### Blocking premature reverse transcription does not rescue defective NC_mutant _reverse transcription kinetics in infected cells

Although the endogenous reverse transcription activity of the NC mutants was essentially wild-type when premature reverse transcription was blocked (Figure 5B, D and 5F), the single-round TZM-bl infectivity of the NC mutants was still reduced compared to wild-type, even after RTI treatment and removal (Figure 4). Because of this disparity, we decided to examine the reverse transcription activities of these mutants during a time course of infection. We generated virus in the presence or absence of RTIs, then PEG-precipitated the viruses (both RTI-treated and untreated samples; Figure 1, left) to remove the RTIs, DNase I treated the inocula (see Methods section), and infected HeLa clone 1022 CD4^(+) ^cells with equivalent amounts of virus, based on exogenous-template RT activities. Cells were then harvested over the time course, total cell DNA was isolated, and vDNA was measured using qPCR. Previously we had reported that using this technique, we were able to see significant differences between wild-type and NC-mutant vDNA kinetic profiles [32].

**Figure 6:**
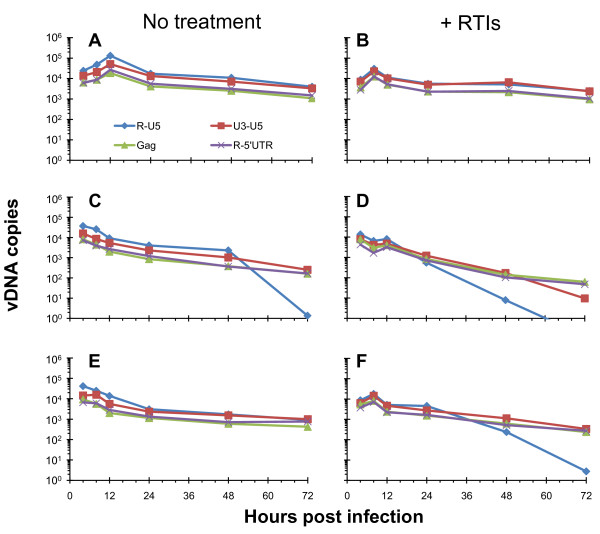
**NC mutant reverse transcription kinetics in cells are altered when premature reverse transcription is blocked**. CD4+ HeLa cells were infected with virus prepared in the absence or presence of RTIs that were subsequently removed using PEG-precipitation (Figure 1, left). These charts display the profile of reverse transcripts over a 72 h time course of infection. Panels A, C, and E show infections from viruses (WT, NC_H23C_, and NC_H44C_, respectively) not treated with RTIs, and panels B, D, and F show infections from viruses (WT, NC_H23C_, and NC_H44C_, respectively) where premature reverse transcription was blocked via RTI treatment. Prior to the infection, all of the virus samples were normalized for RT activity so that equal amounts were used to infect each set of cells. These results are from a representative experiment. The vDNA species measured were normalized for cell equivalents using CCR5 and are indicated at the bottom of panel A. Schematics of the pertinent vDNA target sites are shown at the bottom of Figure 3.

The kinetic profiles of vDNA synthesis during a wild-type infection, with virus prepared without RTIs are shown in Figure 6A. This chart is similar to what we had reported previously--a maximum accumulation of vDNA occurred at 12 h post infection and by 24 h post infection the amounts of R-U5 and U3-U5 are about twice those of Gag and R-5'UTR. In addition, we do not see any evidence for reinfection, although it should be theoretically possible (the cells are CD4+ and the proviral clones are Env^(+)^). However, because Env, Nef, and Vpu can down regulate the CD4 receptor in infected cells [47,48], the lack of reinfection is not necessarily surprising. Figures 6C and 6E show the vDNA profiles after infection with the NC_H23C _and NC_H44C _mutants (without RTI treatment), respectively. As we previously reported, quantities of vDNA at 4 h were similar to wild-type, but unlike wild-type, these were the maximum levels achieved during the entire time course of infection [32].

When we examined vDNA, after infection with wild-type HIV-1 prepared in the presence of RTIs (so that premature reverse transcription was blocked), we saw that the profiles were very similar to those prepared without RTIs (compare panels A and B). The peaks in vDNA syntheses occur at 8 h rather than 12 h, but the ratios of early and late reverse transcripts are the same at all the time points 24 h and later. In addition, in this experiment overall levels of vDNA are about 5-fold lower in viruses prepared with RTIs, although this has no significant effect on single-round infectivity (Figure 4). It is likely that the shift in peak time for vDNA during WT infection is due to infecting cells with higher quantities of virus; when we titrate the amount of virus used to infect cells we see a similar shift in peak vDNA times so that the more virus loaded on the cells results in later vDNA peaks (unpublished observations).

During infections with the NC_H23C _mutant virus prepared with RTIs (Figure 6D) we see a different profile--although initial levels are still the highest, we see an accumulation in reverse transcripts causing a secondary peak at 12 h, then a steeper decrease over the rest of the time course compared to virus prepared without RTIs. In addition, levels of vDNA in the presence of RTIs are about 5-fold lower after infection compared with virus prepared without RTIs (compare Figure 6C and 6D), similar to that observed for the wild-type virus set (Figure 6A and 6B). We see analogous results after infection with NC_H44C _mutant virus (panel F); an accumulation in reverse transcripts with a peak at 8 h post infection, but the overall levels are 5-fold lower than in virus prepared without RTIs (compare panels E and F). The accumulation of peak reverse transcripts in NC mutant viruses prepared with RTIs is likely because these virions do not undergo premature reverse transcription, thus reverse transcription initiates after infection, as with wild-type virus. The fact that the overall levels of vDNA are lower in virus prepared with RTIs, yet the TZM-bl infectivity does not change, indicates that the higher levels of intravirion DNA present in infections with virus prepared without RTIs does not contribute to the infectivity of the virus. The loss of R-U5 products after infection (Figure 6C, 6D and 6F) with the NC mutants is likely due to degradation of the ends of the viral DNA synthesized as well as the lack of integration, which have been previously reported [31,49].

### NC_WT _phenotype is dominant over NC_mutants _and infectivity does not correlate with the extent of premature reverse transcription

**Figure 7:**
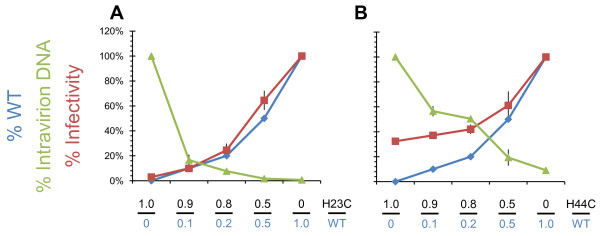
**NC**_**WT **_**phenotype is dominant over NC**_**mutants **_**with no correlation between infectivity and premature reverse transcription**. 293T cells were cotransfected with the indicated ratios of NC_WT _or NC_mutant _proviral plasmids. The percentage of NC_WT _in each transfection is shown in blue. The resulting viruses were harvested and infectious titer in TZM-bl cells was determined (red). The resulting titers were corrected for input virus and are expressed as a percentage of WT titer. The amount of intravirion DNA (R-U5) was also quantified in each sample (green), and these values are expressed as a percentage of intravirion DNA present for each respective NC_mutant _virus (so that 100% mutant is defined as 100% intravirion DNA). The values are the average of 2 separate experiments and the error bars indicate the standard deviations.

We performed the following experiment to test the relationship between intravirion DNA and infectivity by testing whether the NC_WT _or NC_mutant _phenotypes were dominant. We cotransfected cells with different ratios of NC_WT _and NC_mutant _proviral plasmids and examined the virus for infectivity in TZM-bl cells and also measured quantities of intravirion DNA. Figure 7A shows that as the proportion of NC_WT _(blue line) increases relative to NC_H23C_, the amount of intravirion DNA (green line) drops much quicker than the increase in infectivity (red line). However, the increase in infectivity is directly proportional to the increase in the amount of NC_WT_. We see a slightly different result with viruses containing different ratios of NC_WT _to NC_H44C _(Figure 7B) because the decrease in intravirion DNA is more gradual with increasing proportions of NC_WT_. However, in agreement with what was observed with the NC_H23C_:NC_WT _mixtures, the increase in infectivity mirrors the relative amount of NC_WT _present in the virions. The higher overall levels of infectivity observed with the NC_H44C _mutant in this experiment is due to the inherently higher infectious titer of this mutant virus compared to NC_H23C_, (compare red lines between Figures 7A and 7B) which was reported previously [28] and is also apparent in Figure 4. These experiments indicate that the levels of intravirion DNA are independent of the infectivity of these viruses, and that the NC mutations are not dominant over WT HIV-1 with respect to infectivity.

## Discussion

When we observed that the NC_H23C _and NC_H44C _mutations resulted in premature reverse transcription, we did not know if this was a direct, indirect, or unrelated cause of their replication defect [33]. We hypothesized that the presence of intravirion DNA indicates a defective virus. This is based, in part, on observations by Mirambeau and coworkers [50-52] and Cruceanu and coworkers [53] that the mature NC protein (p7) favors binding to single-stranded nucleic acids and binds less tightly to double stranded regions. In addition, Zhang and coworkers reported that when dNTPs were added to extracellular HIV-1 virions, which stimulated reverse transcription, electron micrographs revealed indistinct cores [54]. It seemed reasonable to interpret this core dissolution as being analogous to core uncoating during an infection. Because there is very good evidence that core uncoating is a regulated step during infection [55-59], any event that alters the timing of this could potentially disrupt replication [60,61]. Thus, we wanted to see if the replication defect could be rescued by preventing premature reverse transcription in these NC mutants. To investigate this we developed an experimental system whereby reverse transcription could be reversibly inhibited so that we could examine the effects of blocking the accumulation of high levels of intravirion DNA on infectivity and reverse transcription processes.

In this study, we showed that completely inhibiting premature reverse transcription (Figure 3) did not rescue the single-round infectivity defects associated with the NC mutants (Figure 4). In contrast to this, we found that when premature reverse transcription was blocked, the endogenous reverse transcription kinetics of these mutants could be restored to nearly the wild-type level (Figure 5B, D, and 5F). However, during infection of HeLa CD4+ cells, we did see that the NC_H23C _mutant still showed poor reverse transcription profiles with apparently unstable reverse transcripts (Figure 6D) after RTI treatment. The NC_H44C _mutant showed a profile similar to NC_H23C _virus (compare Figure 6D and 6F). The difference in reverse transcription efficiencies during endogenous reverse transcription versus infection is probably related to the inability of core components to readily diffuse or "uncoat" from the viral core in the endogenous reverse transcription system, as they are maintained within the viral membrane as is discussed below.

The absolute quantities of vDNAs are lower after infections with viruses prepared in the presence of RTIs compared to those prepared without RTIs (Figure 6), however, the infectious titer remains unchanged (Figure 4). This indicates that the amount of intravirion vDNA is irrelevant with respect to infection readout in the TZM-bl assay. Or stated differently, the altered timing of reverse transcription in the NC mutants does not significantly change the infectivity of these viruses using TZM-bl cells as the readout. This conclusion is supported by our experiments with NC_WT _and NC_mutant _mixtures. In these experiments, infectious titer is directly related to the amount of NC_WT _present in the virions, not to the amount of intravirion DNA (Figure 7).

The results from our endogenous reverse transcription time course experiments (Figure 5) are important because we did not see that these NC mutations caused any defects in the kinetics or efficiencies of reverse transcription. Several *in vitro *reverse transcription systems using purified proteins and short defined templates do show reverse transcription defects with these NC mutant proteins [15,62]. The reason for these differences may be as straightforward as protein concentrations. Endogenous reverse transcription occurs within the confines of the virus membrane so that even after 24 h the majority of CA, MA, and NC are still pelletable (data not shown). In addition, if the virus preparations are diluted out prior to endogenous reverse transcription (such as in the time course experiments) the reactions proceed no differently than if the virus is maintained at a high concentration. Because of this, it is likely that during endogenous reverse transcription, the NC protein is maintained at very high concentrations with respect to the viral gRNA. Based on the volume of the conical core of HIV-1 as estimated from cryo-electron tomography [63], and assuming that each virion contains 2000 NC molecules [64], the effective concentration of NC within the core is ~100 mM.

However, despite the fact that the NC mutants have WT endogenous reverse transcription activity upon RTI treatment with subsequent inhibitor removal (Figure 5B, D, F), in the context of an infection, the NC mutants still show defects in reverse transcription compared to wild-type virus (Figure 6). Thus there is something about reverse transcription within the intracellular environment that prevents these NC mutants from productively replicating in a cell. Previously, we had postulated that premature reverse transcription may cause altered uncoating or reverse transcription complex maturation [33], but we now know that premature reverse transcription is not the cause of the replication defect. It is important to point out that the reverse transcripts generated during the infection time course (Figure 6D and 6F) appear more unstable with NC-mutant viruses prepared in the presence of RTIs (premature reverse transcription being blocked), which would indicate that integration is still defective. Although we would like to have examined integration directly using the Alu-LTR qPCR assay as we performed in our previous study [32], the sensitivity of this assay is not amenable to the measurement of integration products with non VSV-G pseudotyped virus infections using HeLa CD4+ cells. However, it is possible that NC is more directly involved during the integration event, as was shown in a previous report using purified integrase and NC in a cell-free, *in vitro *assay [26]. In that study, both of these NC mutants showed a defect in integration compared to wild-type NC. How much this would carry through to infected cells is difficult to say considering that additional viral and cellular protein cofactors are involved [57,59,65].

## Conclusions

We have blocked premature reverse transcription in NC mutant viruses using high levels of RTIs. Upon removal of the inhibitors, the single-round TZM-bl infectivity of these mutants remained the same, independent of whether premature reverse transcription occurred or was blocked. Endogenous reverse transcription assays demonstrated that reverse transcription for these NC mutants displayed wild-type kinetics and efficiencies. However, reverse transcription in the context of an infection was still defective compared to wild-type virus. Cotransfection experiments with various ratios of NC_WT _and NC_mutant _plasmids also failed to show any correlation between intravirion DNA and infectivity. Therefore premature reverse transcription is not the clear-cut cause of the replication defect for these viruses, but is likely a symptom of some other defect in the assembly process.

## Methods

### Chemicals and plasmids

Nevirapine was obtained through the AIDS Research and Reference Reagent Program, Division of AIDS, NIAID, NIH, and handled according to the supplied data sheet (https://www.aidsreagent.org/pdfs/ds4666_004.pdf). Tenofovir (PMPA) was kindly provided by Gilead Sciences, Inc. (Foster City, CA). Construction of the NC_H23C _and NC_H44C _mutants in pNL4-3 (GenBank accession numbers AF324493 and M19921) [66] were previously described [28]. A frameshift mutation was introduced into the *env *of the indicated proviral clones to prevent expression of Env [67]. The plasmid pHCMV-g, which expresses VSV-G [68], was a kind gift from Jane Burns (University of California, San Diego).

### Cell culture and transfections

293T cells [69,70], HeLa CD4+ Clone 1022 (obtained through the AIDS Research and Reference Reagent Program, Division of AIDS, NIAID, NIH: from Dr. Bruce Chesebro https://www.aidsreagent.org/pdfs/ds1109_009.pdf) [71-73], and TZM-bl cells (obtained through the NIH AIDS Research and Reference Reagent Program, Division of AIDS, NIAID, NIH: from Dr. John C. Kappes, Dr. Xiaoyun Wu and Tranzyme Inc. https://www.aidsreagent.org/pdfs/ds8129_010.pdf) [74-78] were maintained as described [28,41]. Transfections were performed using CaPO_4 _co-precipitation as previously described [32,33] with Env^(+) ^virus (Figures 3, 4, 5, 6, 7) or Env^(-)^/VSV-G pseudotyped virus (Figure 2). For RTI treatments we used 50 μM NVP and 1 mM PMPA. Both drugs were added just prior to the addition of DNA precipitates to cells. Cell culture fluids were changed 24 h post transfection and replaced with fresh media containing the same levels of inhibitors. Virus was harvested 48 h post transfection as described [32,33].

### Preparation of virus

Virus was harvested from transfected cells, clarified by low-speed centrifugation and filtration through a 0.22 μm filter as described [32]. We determined the RT activity using the exogenous template RT assay described below. To remove RTIs from virus preparations for subsequent infectivity assays, we added 1/2 volume of ice-cold 30% (wt./vol.) PEG (8000 MW) dissolved in 0.5 M NaCl to the infectious supernatant, then precipitated on ice for at least two h with occasional mixing. Virus-PEG mixtures were then centrifuged at low speed (6,800 × *g*) for 15 min in a prechilled rotor at 4°C. The supernatant was removed by aspiration, and then the pellets were washed with ice-cold 10% (wt./vol.) PEG (1/2 volume of 30% [wt./vol.] PEG (8000) prepared with 1 vol. of Dulbecco's PBS without Ca^2+ ^or Mg^2+^). After the wash supernatant was removed by aspiration, the tubes were spun briefly to collect any remaining supernatant, which was removed by aspiration. The pellets were resuspended in cell culture media and used for infectivity assays (Figure 1, left). For subsequent qPCR analysis of virions and endogenous reverse transcription assays, virus-containing supernatants were treated with DNase I, pelleted through a 20% sucrose pad, then treated with subtilisin and pelleted through another 20% sucrose pad as described [33] (Figure 1, right).

### Infectivity assays

TZM-bl cells in 96-well plates were infected with each PEG-precipitated virus preparation (Figure 1, left) using eleven 3-fold serial dilutions, and the final titer was determined by counting the number of blue colonies in each well and normalizing to the dilution and virus input using exogenous-template RT activity as described [41]. HeLa CD4+ clone 1022 cells were infected with PEG-precipitated virus for 4 h and cells were harvested at the indicated time points. DNA from infected cells was isolated using a Qiagen DNA Blood Mini-Kit, and viral and cellular DNA sequence targets were quantitated using qPCR as described [32].

### Endogenous reverse transcription assays

Virus treated with DNase I and subtilisin (Figure 1, right) was used in the endogenous reverse transcription assay as described [33]. In contrast with the endpoint assays shown previously, we performed a kinetic analysis by following the progression of reverse transcription over a time course. To do this, each DNase-subtilisin treated virus preparation was divided into 7 equal parts. One part was immediately lysed (50 mM Tris, pH 7.4; 10 mM EDTA; 1% (w/v) SDS; 100 mM NaCl; 50 μg/mL yeast tRNA; 100 μg proteinase K), extracted twice each with phenol:chloroform:isoamyl alcohol (25:24:1) and chloroform, and ethanol precipitated. Viral DNA and gRNA were quantitated to assess the initial levels of vDNA and input genomes for the reverse transcription reactions. The other 6 parts were kept on ice while endogenous reverse transcription buffer was added to each tube (final composition after addition to virus sample, 50 mM Tris-HCl, pH 8.0, 2 mM MgCl_2_, 10 mM dithiothreitol, 25 μM [each] dNTPs). All samples were placed at 37°C simultaneously, and at the indicated times one part was collected, immediately lysed (as above) and viral DNA was quantitated to determine progression of reverse transcription.

### Exogenous-template reverse transcriptase assays

Virus containing supernatants (0.75 mL) were clarified by low speed centrifugation and/or filtration and mixed with 0.375 mL of 30% (wt./vol.) PEG (8000 MW) dissolved in 0.5 M NaCl. Samples were stored overnight at 4°C, precipitates were collected by centrifugation at top speed in a microfuge, supernatants were removed and pellets were resuspended in 37.5 μL of 50 mM Tris, 100 mM NaCl, 1 mM EDTA, 2% (vol./vol.) fetal bovine serum, pH 7.5. The resuspended virus (5 μL) was assayed in a total volume of 25 μL containing 50 mM Tris, 100 mM NaCl, 6 mM MgCl_2_, 10 mM dithiothreitol, 4 μg/mL oligo-dT_17 _(Invitrogen, Carlsbad, CA), 40 μg/mL poly-rA (The Midland Certified Reagent Company, Inc., Midland, TX), 0.01 μCi [α-^32^P]-TTP (3000 Ci/mmol; Perkin Elmer Life Sciences, Waltham, MA) and 0.25% (vol./vol.) Nonidet P-40.

Samples were incubated at 37°C for 3 h, then 5 μL were spotted onto a DEAE Filtermat for the 1450 MicroBeta counter (Perkin Elmer Cat. No.: 1450-5222) and allowed to dry. Filters were washed 3× in 250 mL of 0.3 M NaCl, 0.03 M sodium citrate, pH 7.0, for 15 min each, then rinsed twice in 10 mL of 95% (vol./vol.) ethanol (1 min each) and allowed to dry. The filtermats were counted using a ^32^P-Filtermat Cassette (Perkin Elmer cat. no.: 1450-118) in a Wallac 1450 Microbeta, 6-detector, liquid scintillation counter (Perkin Elmer).

### q PCR for vDNA and qRT-PCR for gRNA

Primers and probes were used for quantitation of gRNA and vDNA using a Stratagene Mx3000P instrument (Agilent Technologies, Santa Clara, CA). All of the primers, probes, and PCR conditions used have been described [32,33]. The targets monitor progression of 4 discrete steps of reverse transcription including minus-strand strong-stop synthesis (R-U5), minus-strand transfer product (U3-U5), late minus-strand synthesis (Gag), and plus-strand transfer product (R-5'UTR). For gRNA determination the primers and probes for *gag *detection were used (see bottom of Figure 3 for schematics of the pertinent vDNA target sites). CCR5 DNA copies were used to normalize vDNA for cell recovery in infections (Figure 6) as described [32].

## Competing interests

The authors declare they have no competing interests.

## Authors' contributions

JAT and RJG designed the experiments, performed the experiments, analyzed data, and wrote the paper. TLS performed cell culture and assisted with the transfections and infectivity analyses. All authors have read and approved the final version of this manuscript.
